# Interaction of aldehydes derived from lipid peroxidation and membrane proteins

**DOI:** 10.3389/fphys.2013.00242

**Published:** 2013-09-04

**Authors:** Stefania Pizzimenti, Eric Ciamporcero, Martina Daga, Piergiorgio Pettazzoni, Alessia Arcaro, Gianpaolo Cetrangolo, Rosalba Minelli, Chiara Dianzani, Alessio Lepore, Fabrizio Gentile, Giuseppina Barrera

**Affiliations:** ^1^Dipartimento di Scienze Cliniche e Biologiche, Università di TorinoTorino, Italy; ^2^Dipartimento di Medicina e Scienze della Salute, Università del MoliseCampobasso, Italy; ^3^Istituto di Biochimica delle Proteine del Consiglio Nazionale delle RicercheNapoli, Italy; ^4^Dipartimento di Scienza e Tecnologia del Farmaco, Università di TorinoTorino, Italy; ^5^Dipartimento di Medicina Molecolare e Biotecnologie Mediche – Università degli Studi di Napoli Federico IINapoli, Italy

**Keywords:** lipid peroxidation, aldehydes, membrane proteins, human diseases, signal transduction

## Abstract

A great variety of compounds are formed during lipid peroxidation of polyunsaturated fatty acids of membrane phospholipids. Among them, bioactive aldehydes, such as 4-hydroxyalkenals, malondialdehyde (MDA) and acrolein, have received particular attention since they have been considered as toxic messengers that can propagate and amplify oxidative injury. In the 4-hydroxyalkenal class, 4-hydroxy-2-nonenal (HNE) is the most intensively studied aldehyde, in relation not only to its toxic function, but also to its physiological role. Indeed, HNE can be found at low concentrations in human tissues and plasma and participates in the control of biological processes, such as signal transduction, cell proliferation, and differentiation. Moreover, at low doses, HNE exerts an anti-cancer effect, by inhibiting cell proliferation, angiogenesis, cell adhesion and by inducing differentiation and/or apoptosis in various tumor cell lines. It is very likely that a substantial fraction of the effects observed in cellular responses, induced by HNE and related aldehydes, be mediated by their interaction with proteins, resulting in the formation of covalent adducts or in the modulation of their expression and/or activity. In this review we focus on membrane proteins affected by lipid peroxidation-derived aldehydes, under physiological and pathological conditions.

## Introduction: lipid peroxidation-derived aldehydes

Reactive intermediates produced under conditions of oxidative stress cause the oxidation of polyunsaturated fatty acids (PUFAs) in membrane lipid bilayers, leading eventually to the formation of aldehydes (Esterbauer et al., 1991). Among these, the most abundant aldehydes are 4-hydroxy-2-nonenal (HNE) and malondialdehyde (MDA), while acrolein is the most reactive one (Esterbauer et al., 1991). HNE is the lipoperoxidation product which has displayed the highest biological activity and, for this reason, has been most intensively studied. On the other hand, acrolein, which is the most electrophylic compound, has received less attention, because it is scarcely represented among lipoperoxidation products. Both acrolein and HNE are α,β-unsaturated electrophilic compounds, which preferentially form 1,4-Michael type adducts with nucleophiles, such as proteins and DNA. Even though MDA shows little reactivity under physiological conditions, at low pH its reactivity increases, when beta-hydroxyacrolein becomes the predominant species and, analogously to acrolein and HNE, it can form 1,4-Michael type adducts with nucleophiles (Esterbauer et al., 1991). Even though it was demonstrated that MDA does not react with glycine and GSH, and reacts slowly with cysteine (Esterbauer et al., 1991) under physiological conditions, cellular proteins are much more readily modified by MDA (Chio and Tappel, 1969).

Due to the high chemical reactivity of aldehydes, mammals have evolved a battery of enzymes which convert these compounds to less reactive chemical species. The main reactions of aldehydes are the adduction with glutathione (GSH), which can either occur spontaneously or be catalyzed by glutathione S-transferases (GSTs), the reduction to alcohol by aldo–keto reductases (AKRs) or alcohol dehydrogenase and the oxidation to acid by aldehyde dehydrogenases. The metabolism of aldehydes has been reviewed in excellent mode by Esterbauer and collaborators (1991). More recent reviews were focused on the biochemistry of lipid peroxidation products (Guéraud et al., 2010) and acrolein biotransformation (Stevens and Maier, 2008). The catabolic rates of the various aldehydes contribute, together with their rates of production from lipid peroxidation, in determining their steady-state intracellular concentrations. At high concentrations, all these aldehydes were found to play a role in the toxic effects of lipid peroxidation. Aldehyde toxicity is mainly due to the alterations of several cell functions, which mostly depend on the formation of covalent adducts with cellular proteins (Grimsrud et al., 2008). Due to their amphiphilic nature, aldehydes can easily diffuse across membranes and can covalently modify any protein in the cytoplasm and nucleus, far from their site of origin (Negre-Salvayre et al., 2008). Similarly, the aldehydes formed outside the cells (i.e., in a site of inflammation or in plasma), can react with adjacent cells, even in cases when they are not primary sites of lipid peroxidation. In the latter instance, plasma membrane proteins represent the first targets for adduct formation. Exogenous or endogenous aldehydes can react also with nuclear proteins, thus modulating protein expression through their reaction with transcription factors or other regulatory elements (Jacobs and Marnett, 2010). The targets of lipid peroxidation-derived aldehydes are cell-type specific and dependent both on the pattern of proteins expressed by the cell and the aldehyde concentration. Moreover, the modification of a specific protein can have different biological consequences, in relation to its specific cell function. However, at low concentration, HNE in particular can play an important role in signal transduction and exert antiproliferative and anti-invasive actions toward cancer cells, by interfering with the modulation of gene expression via the formation of protein and/or DNA adducts (Gentile et al., 2009; Barrera, 2012).

**Figure 1:**
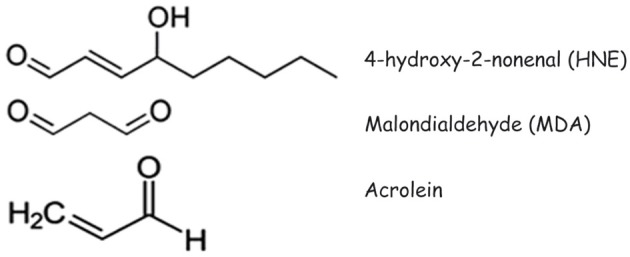
**Structures of 4-hydroxy-2-nonenal (HNE), malondialdehyde (MDA) and acrolein**.

The presence of aldehyde-protein adducts has been demonstrated in a wide range of physiological and pathological conditions. Those among the latter in which aldehyde-protein adducts, in particular HNE-protein adducts, have been most intensively studied are neurodegenerative diseases and atherosclerosis. Recently, a role has emerged for aldehyde-protein adducts in autoimmune diseases, since the covalent alteration of protein structure can bring about a sufficient modification of a self antigen for it to break the immunological tolerance of autoreactive T and/or B cells. In the following sections, we shall examine the mechanisms of formation of aldehyde-protein adducts and the main biological consequences of the formation of aldehyde adducts with membrane proteins in neurodegenerative diseases, atherosclerosis, autoimmune diseases and in relation with the functions played by cell proteins at the plasma membrane level. The chemical structures of HNE, MDA and acrolein are illustrated in Figure 1

## Characteristics of aldehydes and their protein adducts

### 4-hydroxynonenal (HNE)

4-Hydroxynonenal (HNE) is an aldehyde highly represented among the products of lipid peroxidation, which displays high biological activity. This aldehyde has three main functional groups: the aldehyde group, the C=C double bond and the hydroxyl group, which can participate, alone or in sequence, in chemical reactions with other molecules (Esterbauer et al., 1991). Due to its strong hydrophobic nature, HNE is mostly associated with the membranes where it is produced, but it can also diffuse to different cellular compartments (Butterfield and Stadtman, 1997). HNE is a highly electrophilic molecule that easily reacts with glutathione, proteins and, at higher concentration, with DNA. HNE forms adducts with three different amino acyl side chains, namely of Cys, His, and Lys residues, via Michael addition either to thiol (−SH) or to amino (−NH_2_) groups. Cys residues display the highest reactivity with HNE, even though Cys residues are not always the preferential targets of HNE, because the tertiary structure of the protein can condition their accessibility and, therefore, their reactivity toward exogenous chemicals. No reaction of HNE was detected with Glu (Doorn and Petersen, 2003). Besides by the simple formation of Michael adducts to lysyl, histidyl, and cysteinyl residues (Esterbauer et al., 1991), HNE can modify protein structure through Schiff base formation with lysyl residues, leading to pyrrole formation (Sayre et al., 1996). In addition, HNE modification can result in the cross-linking of two lysyl residues through reversibly formed Schiff base Michael adducts (Parola et al., 1999; Xu et al., 1999), as well as irreversibly formed 2-hydroxy-2-pentyl-1,2-dihydropyrrol-3-one iminium moieties (Parola et al., 1999; Dianzani, 2003; Barrera et al., 2008). The target proteins of HNE adduct formation *in vitro* and *in vivo* have been reviewed in great detail by Poli et al. (2008).

HNE has been detected *in vivo* in several pathological conditions characterized by increased lipid peroxidation, including inflammation, atherosclerosis, chronic degenerative diseases of the nervous system, and chronic liver diseases (Moreau et al., 2005).

### Acrolein

Acrolein is a little aldehyde with three carbon atoms and a double bond. Besides being formed endogenously during lipid peroxidation, this aldehyde is inhaled with cigarette smoke and is present in cooked oils and other foods (Stevens and Maier, 2008). Acrolein is the strongest electrophile in the α,β-unsaturated aldehyde series; its reaction with the thiol group of cysteine was about 110–150 times faster than that of HNE (Esterbauer et al., 1991; Witz, 1997). The toxicity of acrolein is related to its ability to deplete glutathione (Kehrer and Biswal, 2000), and to form DNA and protein adducts (Esterbauer et al., 1991; Sanchez et al., 2005; Feng et al., 2006). Potential targets of acrolein in proteins include the side chains of cysteinyl, histidyl, and lysyl residues, as well as free N-terminal amino groups (Cai et al., 2009). Cysteine is widely accepted as the most likely site of acrolein adduct formation. The sulfhydryl group of a cysteinyl residue is the most reactive nucleophile in proteins and the thiol adducts with acrolein are considerably more stable than the adducts formed by other α,β-unsaturated aldehydes (Esterbauer et al., 1991; Witz, 1997). Cysteinyl residues are located at the active sites of several proteins and are often involved in the catalytic activity of enzymes, thus the formation of acrolein-cysteine adducts has broad functional implications. It has been reported that the modification of cysteinyl residues by acrolein leads to the inactivation of enzymes, such as aldose reductase (Srivastava et al., 1999) and protein tyrosine phosphatase 1B (Seiner et al., 2007). However, no cysteine adducts of acrolein have been identified *in vivo*. Other Authors have shown that acrolein generated during lipid peroxidation may primarily react with histidyl residues of proteins, to form Nτ-(3-propanal)-histidine and that acrolein-histidine is the major adduct formed with proteins in *in vitro* studies (Maeshima et al., 2012).

Elevated plasma concentrations of acrolein are detected in patients with chronic renal failure, and the abundance of the proteins adducts of acrolein is increased in tissues obtained from patients with Alzheimer's disease, Parkinson's disease, atherosclerosis and chronic obstructive lung disease (Uchida et al., 1998a; Shamoto-Nagai et al., 2007; Stevens and Maier, 2008; Moretto et al., 2012).

### Malondialdehyde (MDA)

Malondialdehyde (MDA) is widely used as a marker for the peroxidation of ω 3 and ω 6 fatty acids, measured by the chemical determination of thiobarbituric acid reactive substances (TBARS) (Negre-Salvayre et al., 2010), although the latter provides an incomplete perspective, as MDA derives from the decomposition of only certain lipid peroxidation products and is neither the sole end product, nor one of lipid peroxidation only (Halliwell and Whiteman, 2004). At neutral pH, MDA is present as enolate anion, with low chemical reactivity (Esterbauer et al., 1991). Nevertheless, it is able to interact with nucleic acid bases to form several different adducts (Marnett, 1999). MDA has been reported to react *in vivo* with primary amines, to form Nε-(2-propenal) lysine and generate lysine-lysine cross-links with 1-amino-3-iminopropene and pyridyledihydropyridine type bridges (Uchida, 2000). These reaction products have been detected in Apo B fractions of oxidized lipoproteins (LDL) and are thought to be involved in the impaired interaction of modified lipoproteins with macrophages (Palinski et al., 1994). Mooradian and coworkers have reported that protein glycosylation and the presence of acetaldehyde enhance MDA modification of proteins (Mooradian et al., 1996, 2001). Moreover, MDA and acetaldehyde can form stable adducts (MAA) (Tuma et al., 1996) and can react covalently and synergistically with proteins, forming MAA–protein adducts. The latter can be pro-inflammatory and pro-fibrogenic and are capable of inducing strong immune responses (Tuma, 2002).

### Phosphatidyl γ-hydroxyalkenals (PC-HAs)

**Figure 2:**
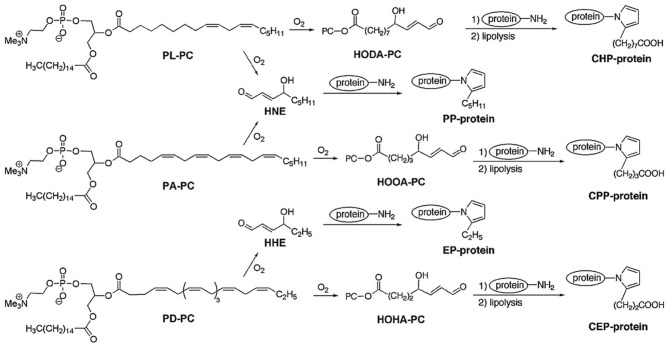
**Structures and reactions of phosphatidylcholine γ-hydroxyalkenals (PC-HAs)**. In addition to 2-pentylpyrrole-modification of proteins by the electrophilic addition of HNE to ε-amino lysyl groups, carboxyalkylpyrrole-modified proteins are also formed by adduct formation with other γ-hydroxyalkenals (mirror images of esterified HNE) also formed, at the time of HNE formation, from the oxidation of PUFA-containing phospholipids. Legend: HHE, 4-hydroxy-2-hexenal; HNE, 4-hydroxy-2-nonenal; PL-PC, 1-palmitoyl-2-linoleoyl-*sn*-glycero-3-phosphocholine; PA-PC, 1-palmitoyl-2-arachidonoyl-*sn*-glycero-3-phosphocholine; PD-PC, 1-palmitoyl-2-docosahexanoyl-*sn*-glycero-3-phosphocholine; HODA-PC, 9-hydroxy-12-oxo-10-dodecenoyl-phosphatidylcholine (this compound may also derive from 1-linoleoyl-2-arachidonoyl-*sn*-glycero-3-phosphocholine, LA-PC); HOOA-PC, 5-hydroxy-8-oxo-6-octenoyl-phosphatidylcholine; HOHA-PC, 4-hydroxy-7-oxo-5-heptenoyl-phosphatidylcholine; PP-protein, 2-pentylpyrrole-modified protein; EP-protein, 2-ethylpyrrole-modified protein; CPP-protein, carboxypropylpyrrole-modified protein; CEP-protein, carboxyethylpyrrole-modified protein [Reprinted with permission from Salomon et al. (2011)].

Phosphatidylcholine γ-hydroxyalkenals (PC-HAs) are the most abundant and biologically relevant compounds in the class of γ-hydroxyalkenal phospholipids, deriving from the peroxidation of polyunsaturated fatty acids (PUFAs) esterified to phosphoglycerides at the *sn*-2 position of phosphatidylcholine (PC). β-Scission of an alkoxyl radical derived from dihydroperoxide produces two γ-hydroxy-α,β-unsaturated aldehydes, i.e., a methyl-terminal HNE molecule and a mirror image of HNE, still esterified to PC (namely, 9-hydroxy-12-oxo-10-dodecenoic acid [HODA] or its PC ester from linoleate and 5-hydroxy-8-oxo-6-octenoic acid [HOOA] or its PC ester from arachidonate). Because they possess a γ-hydroxy-α,β-unsaturated terminal aldehyde like HNE, PC-hydroxyalkenals are expected to form Michael adducts with primary amino groups of lysyl residues and thiol groups of cysteinyl residues, as well as pentylpyrrole adducts, incorporating the ε-amino groups of lysyl residues (Figure 2). γ-Hydroxyalkenal phospholipids contribute strongly in the pathogenesis of the atherosclerotic disease. ω-Carboxyalkylpyrrole modifications of proteins, after lypolysis of intermediate phospholipid adducts, are of pathogenetic importance in age-related macular degeneration, autism and cancer, and promote wound healing. In regard, the reader is referred to the excellent reviews by Salomon et al. (2011), and Salomon and Gu (2011).

## Aldehyde-protein adducts in neurodegenerative diseases

Central nervous system (CNS) is one of the major targets of lipid peroxidation. The brain is highly sensitive to oxidative stress because it consumes about 20–30% of inspired oxygen and contains high levels of PUFAs. In particular, high levels of the markers of lipid peroxidation have been found in brain tissues and body fluids in several neurodegenerative diseases, including Alzheimer's disease (AD), Parkinson disease (PD), amyotrophic lateral sclerosis (ALS), Huntington disease (HD) and Down syndrome (DS) (Sajdel-Sulkowska and Marotta, 1984; Butterfield et al., 2010; Ruiperez et al., 2010; Lee et al., 2011; Shichiri et al., 2011). We focus here on the adducts of lipid peroxidation-derived aldehydes with protein targets whose oxidative modifications might be relevant for the neuronal dysfunctions observed in Alzheimer's disease.

### Alzheimer's disease (AD)

Oxidative damage occurs in early stages of Alzheimer's disease (AD) (Butterfield et al., 2006a; Reed et al., 2008, 2009a; Mangialasche et al., 2009). Several Authors, using redox proteomic approaches, have undertook the task of compiling inventories of cellular proteins modified as a consequence of increased oxidation, glycoxidation, or lipoxidation in the course of oxidative and/or nitrosative stress (Pamplona et al., 2005; Butterfield et al., 2006b; Newman et al., 2007; Reed et al., 2008, 2009a,b; Perluigi et al., 2009). Other searches focused upon proteins modified by tyrosine nitration (Castegna et al., 2003; Sultana et al., 2006a; Reed et al., 2008) and S-glutathionylation (Newman et al., 2007). A number of comprehensive reviews are available (Mangialasche et al., 2009; Martínez et al., 2010; Reed, 2011; Sultana et al., 2012). Like in other neurodegenerative diseases with proteinaceous deposits, vulnerable proteins could be assigned to a few distinct functional groups with crucial roles in plasma membrane ion and nutrient transport, energy metabolism (glycolysis, mitochondrial electron transport and oxidative phosphorylation), cell signaling, cytoskeletal organization, antioxidant defenses, cellular stress responses, protein synthesis, signal transduction, and regulation of neurotransmission (Table 1).

**Table 1:** **HNE-protein adducts detected in Alzheimer's disease, in relation with disease progression^a^**.

**Protein**	**AD stage^a^**	**Function**	**References**
Aldolase	PAD, LAD	Energy metabolism	Perluigi et al., 2009
Triose phosphate isomerase (TPI)	EAD	Energy metabolism	Reed et al., 2009a
Phosphoglycerate kinase (PGK)	MCI	Energy metabolism	Reed et al., 2008
α-Enolase (non neural enolase, ENO1)^b^	MCI, EAD, LAD	Energy metabolism	Reed et al., 2008, 2009a; Perluigi et al., 2009
Pyruvate kinase (PK) M2 isoform	PAD, MCI	Energy metabolism	Reed et al., 2008
Lactate dehydrogenase B (LDHB)	MCI	Energy metabolism	Reed et al., 2008
Aconitase	PAD, LAD	Energy metabolism, mitochondrial function	Perluigi et al., 2009
Malate dehydrogenase, mitochondrial	EAD	Energy metabolism, mitochondrial function	Reed et al., 2009a
ATP synthase α subunit	PAD, MCI, EAD, LAD	Energy metabolism, mitochondrial function	Reed et al., 2008, 2009a; Perluigi et al., 2009
Mn Superoxide dysmutase (SOD2)	EAD, LAD	Mitochondrial function, antioxidant defense	Perluigi et al., 2009; Reed et al., 2009a
Carbonyl reductase 1	MCI	Antioxidant defense	Reed et al., 2008
Peroxiredoxin VI (Phospholipase A2)	LAD	Antioxidant defense	Perluigi et al., 2009
Heme oxygenase 1 (HO-1)	MCI, LAD	Antioxidant defense	Sultana et al., 2012
70-kDa heat shock protein (HSP70)	MCI	Stress response	Reed et al., 2008
Pleckstrin homology-like domain, family A, member 2 (IPL)		Signal transduction	Reed et al., 2008
β-Actin	MCI	Cytoskeleton	Reed et al., 2008
α-Tubulin	LAD	Cytoskeleton	Perluigi et al., 2009
Elongation factor Tu (EF-Tu)	PAD, MCI	Protein synthesis	Reed et al., 2008
Initiation Factor α (eIFα)	MCI	Protein synthesis	Reed et al., 2008
Glutamine synthetase	LAD	Excitotoxicity	Perluigi et al., 2009
Neuropolypeptide h3	PAD, MCI	Neuronal communication	Reed et al., 2008
Collapsin response mediated protein 2 (CRMP-2)^c^	EAD, LAD	Neuronal communication	Perluigi et al., 2009; Reed et al., 2009a

a *Clinical stages of Alzheimer's disease (AD) progression, in chronological order: PAD, preclinical AD; MCI, mild cognitive impairment; EAD, early stage AD; LAD, late stage AD*.

b *Integral or perypheral membrane proteins of plasma or organelle membranes are underlined*.

c *Also known as dihydropirimidinase-related protein 2 (DRP-2)*.

#### HNE-amyloid β peptide adducts

Amyloid β (Aβ) peptide is the major protein component of amyloid plaques and one of the main components of neurofibrillary tangles (NFTs), hallmarks of AD. This molecule is a 40-to-42-amino acid peptide derived from the integral membrane Amyloid Precursor Protein (APP), through sequential proteolytic cleavages by β-secretase (BACE) and γ-secretase (Hardy and Selkoe, 2002). HNE can react directly with the Aβ peptide. This process was reported to exacerbate the formation of toxic Aβ-dependent diffusible oligomers and insoluble aggregates, which, in turn, enhanced oxidative stress, fromation of lipid peroxidation products, such as HNE, and Aβ oligomerization and toxicity (Siegel et al., 2007). HNE modified the three histidyl residues of Aβ, so that HNE-modified Aβ molecules had increased affinities for membrane lipids and adopted a similar conformation as mature amyloid fibrils (Murray et al., 2007; Liu et al., 2008).

#### HNE-α-enolase adducts

α-Enolase (non neural enolase, ENO1) is a multiform, multifunctional protein. In the cytoplasm, it is a 48-kDa enzyme, catalyzing 2-phospho-D-glycerate dehydration to phosphoenolpyruvate. At the cell surface of neutrophils, B and T cells, monocytes, epithelial, endothelial cells and neurons, it serves as a plasminogen receptor, involved in fibrinolysis (Pancholi, 2001) and in neutrophil and monocyte recruitment (Busuttil et al., 2004; Wygrecka et al., 2009). Binding to α-enolase protects plasmin from inactivation by α 2-antiplasmin (Bergman et al., 1997). Alternative translation of α-enolase mRNA produces a 37-kDa protein, preferentially located in the nucleus (myc Binding Protein-1, MBP-1), with *c-myc* gene promoter-binding and tanscription repressing activity (Feo et al., 2000; Subramanian and Miller, 2000). In addition, α-enolase was reported to be a hypoxic stress protein (Graven and Farber, 1998). A regulatory circuit between c-myc, MBP-1, and α-enolase was described, connecting cell energy metabolism and proliferation (Sedoris et al., 2007). Redox proteomic studies identified α-enolase as a target of oxidative modification in all stages of Alzheimer's disease, undergoing the formation of carbonyl groups (Castegna et al., 2002; Butterfield et al., 2006b; Sultana et al., 2006b), MDA adducts (Pamplona et al., 2005), 4-HNE adducts (Reed et al., 2008, 2009a; Perluigi et al., 2009), tyrosine nitration (Castegna et al., 2003; Sultana et al., 2006a; Reed et al., 2009b) and S-glutathionylation (Newman et al., 2007). A typical metabolic feature of AD is the reduced rate of glucose metabolism, as seen in positron-emission tomography with 2-[^18^F]fluoro-2-deoxy-D-glucose (FDG/PET) (de Leon et al., 2001). Despite compensatory increases of α-enolase expression in AD (Sultana et al., 2007) and even though enzymatic activity was not assayed, it was suggested that the loss of function associated with the oxidative modifications of α-enolase might render neurons prone to apoptosis, by dysrupting their energy metabolism (Sultana et al., 2012). As peptide Aβ (1-42), by aggregating in cross-β-structured fibrils, with a similar conformation as fibrin peptides, could substitute for fibrin in the activation of tissue plasminogen activator (tPA) (Kingston et al., 1995), and because plasmin cleaved peptide Aβ 1-40 into a truncated form, with potent stimulatory activity toward tPA (VanNostrand and Porter, 1999), it was proposed that the loss of plasminogen-binding activity of HNE-α-enolase might foster apoptosis in AD, by hindering Aβ peptide degradation (Sultana et al., 2012).

Such a scenario is supported by a functional study of HNE-α-enolase adducts in HL-60 leukemic cells (Gentile et al., 2009). α-Enolase was among a few proteins recognized by anti-histidine-HNE antibodies, after 15 min of exposure to 1–10 μ M HNE. HNE–α-enolase adducts were detected early on the surface of HL-60 cells, indicating a high degree of α-enolase exposure. HNE treatment did not alter α-enolase expression or enzymatic activity. The low-level expression of an anti-α-enolase Ab-reactive 37-kDa peptide, possibly corresponding to MBP-1, did not vary after HNE treatment. The main functional alteration of HNE-α-enolase concerned its plasminogen-binding ability. Treatment with 1 μ M HNE strongly inhibited plasminogen binding to α-enolase at the cell surface and consequently reduced HL-60 cell adhesion to human umbilical venous cells (HUVECs), suggesting that HNE and other inhibitors of plasminogen binding to α-enolase may be of use in the control of tumor invasion. α-Enolase emerged from this study as a protein most susceptible to HNE adduct formation, in keeping with various proteomic studies, in the context of neurodegeneration, cited in section Alzheimer's Disease (AD), which pinpointed a limited number of protein targets of oxidative modification, including α-enolase, grouped in selected functional subsets (Martínez et al., 2010; Reed, 2011; Sultana et al., 2012). Some of these proteins targets were also identified as autoantigens frequently recognized by autoantibodies in autoimmune diseases. α-Enolase, in particular, was recurrently indicated as a novel autoantigen in systemic lupus erythematosus (SLE), systemic sclerosis (SSc) (Moscato et al., 2000; Pratesi et al., 2000; Mosca et al., 2006; Bussone et al., 2011), SSc with interstitial lung fibrosis (Terrier et al., 2010), rheumatoid arthritis (Goëb et al., 2009; Saulot et al., 2002), mixed cryoglobulinemia (MC) with nephropathy (Sabbatini et al., 1997; Moscato et al., 2000; Pratesi et al., 2000), pulmonary arterial hypertension (Bussone et al., 2012), giant-cell arteritis (Régent et al., 2011), Behçet's disease (Lee et al., 2003) and inflammatory bowel disease (IBD) (Roozendaal et al., 1998). Anti-α-enolase autoantibodies isolated from patiens with SLE, SSc and MC recognized membrane-associated α-enolase and inhibited plasminogen binding to it (Moscato et al., 2000). It is tempting to speculate that the high susceptibility of α-enolase to modification by HNE and other aldehydes might be instrumental for its involvement in autoimmunity as an oxidatively modified self antigen, capable of breaking the immunological tolerance of autoreactive T and B cells. This is in keeping with the identification of α-enolase among the proteins undergoing carbonyl addition and HNE adduction in heart homogenates and cardiomyocytes oxidized *in vitro* with 4-HNE or H_2_O_2_. Oxidative modifications correlated with increased recognition of α-enolase by serum antibodies of rodents and humans affected with Chagas' disease, which is characterized by increased production of ROS of inflammatory and mitochondrial origin (Dhiman et al., 2012).

#### HNE adducts with other neuronal enzymes, transporters, and receptors

Inducible ***heme oxygenase 1 (HO-1)*** catalyzes heme conversion to biliverdin-IXa, which is further reduced to antioxidant bilirubin-IXa (Mancuso and Barone, 2009). The expression of the *HO-1* gene is redox-regulated by an antioxidant responsive element in its promoter. Activation of HO-1 contributes to the adaptive response to oxidative stress in AD (Poon et al., 2004). Increased levels of HO-1 were observed in association with neurofibrillary tangles (NFTs) and senile plaques (Takeda et al., 2000) and in hippocampal neurons of AD patients, together with increases of serine phosphorylation, tyrosine nitration and 4-HNE modification of HO-1, as though adaptive increases in HO-1 expression and activation were counteracting the structural and functional impairment of HO-1, via tyrosine nitration and HNE-HO-1 adduct formation.

***Collapsin response mediator protein 2 (CRMP2)***. Participates in axon guidance and synapse maturation, by mediating the transduction of reelin (Yamashita et al., 2006) and semaphorin 3A signals (Uchida et al., 2009). Sultana et al. (2012) has proposed that the HNE-CRMP2 adducts (Reed et al., 2008; Perluigi et al., 2009) might be of pathogenic importance for neurite shortening and the loss of synapses, early features of AD (Hensley et al., 2011; Scheff et al., 2011), and that Aβ peptide-induced oxidation of peptidylprolyl cis/trans isomerase (Pin1) (Butterfield et al., 2006b; Sultana et al., 2006b) may be responsible for the dysregulation of glycogen synthase kinase-3β (GSK-3β) and cyclin-dependent kinase 5 (CDK5) and for the hyperphosphorylation of tau proteins and of colocalized CRMP2 within NFTs (Williamson et al., 2011).

Reduced glucose utilization and energy production (Rhein and Eckert, 2007) are early occurrences in AD. They may be explained by the reported formation of HNE adducts with neuronal ***glucose transporter GLUT3*** in rat hippocampal neurons (Mark et al., 1997a) and with the ***mitochondrial ATP synthase*** ***α subunit*** in human AD brains (Reed et al., 2008; Perluigi et al., 2009; Terni et al., 2010). Decreased levels of ATP synthase activity were also reported in AD (Schagger and Ohm, 1995). Soluble Aβ peptide oligomers were responsible for electron-transport chain dysruption, enhanced ROS generation, mitochondrial fragmentation, and synaptic damage (Reddy et al., 2010), as well as for enhanced HNE production (Mark et al., 1997b). ATP synthase α subunit was colocalized with CRMP2 within NFTs (Sergeant et al., 2003). In AD brains, LDL receptor-related protein 1 (LRP-1), a membrane receptor involved in Aβ peptide removal, was also covalently modified by HNE, which might contribute to the extracellular deposition of amyloid substance (Owen et al., 2010).

#### Acrolein-protein adducts

Acrolein is neurotoxic *in vitro*. Moreover, in Alzheimer's brains, high levels of acrolein were detected in hippocampus and temporal cortex, where oxidative stress is high (Dang et al., 2010). Thus, several studies addressed the mechanism of acrolein neurotoxicity. Dang and coworkers (2010) showed that, in neuronal primary cultures of hippocampal cells, acrolein exerted more toxic effects than HNE. This might depend on the higher reactivity of acrolein, which was an initiator of oxidative stress by forming adducts with cellular nucleophilic groups in proteins, lipids, and nucleic acids. Indeed, it was documented that in synaptosomal proteins, exposed to high concentrations of acrolein, a loss of thiol group content occurred, due to Michael adduct formation between acrolein and thiol groups of proteins (LoPachin et al., 2007). Moreover, such adduct formation led also to protein cross-linking. Based on the cited evidence, LoPachin et al. (2002, 2003) proposed that nerve terminals were the primary sites of acrolein action and that synaptic dysfunction was a necessary step in the production of neurotoxicity. In support of this hypothesis, *in vivo* and *in vitro* studies showed that exposure to acrolein was associated with reduced presynaptic neurotransmitter release. This effect involved inhibition of key proteins, which regulate membrane–vesicle fusion, such as N-ethylmaleimide-sensitive fusion protein (NSF) and synaptosomal-associated protein of 25 kDa (SNAP-25) (Barber and LoPachin, 2004; LoPachin, 2004). Acrolein also inhibited presynaptic membrane neurotransmitter uptake and vesicular storage *in vivo* and *in vitro* (LoPachin et al., 2006; LoPachin, 2004). Proteomic analyses showed that these dysfunctions were associated with the formation of adducts with the ***dopamine transporter*** and ***v-ATPase***, respectively Barber and LoPachin, 2004; LoPachin, 2004; LoPachin et al., 2007; Barber et al., 2007. *In vitro* studies showed that acrolein and HNE disrupted synaptosomal membrane protein conformation and phospholipid asymmetry (Subramaniam et al., 1997; Pocernich et al., 2001; Castegna et al., 2004), reduced glutamate uptake and GLUT3-mediated glucose transport in synaptosomes and cultured nerve cells (Keller et al., 1997a,b; Lovell et al., 2000), reduced respiration and induced oxidative stress in synaptosomal mitochondria (Humphries et al., 1998; Morel et al., 1999; Picklo et al., 1999; Picklo and Montine, 2001; Luo and Shi, 2005; Raza and John, 2006), inhibited membrane Na^+^ and Ca^2+^ ion pumps and dysrupted ion regulation in cultured nerve cells (Keller et al., 1997b; Mark et al., 1997b).

#### MDA-protein adducts

A largely coincident protein repertoire as the one delineated by anti-HNE antibodies was compiled by the immunochemical detection of Nε-MDA-lysine. It included: α- (non neural) and γ-enolase (neural), glutamic acid dehydrogenase I, creatin kinase B chain (CKB), ubiquinol-cytochrome c reductase complex core protein I, ATP synthase β subunit, glutathione synthase (GS), 60-kDa heat shock protein (HSP-60), guanine nucleotide-binding protein G(I)/G(S)/G(T) β subunit 2 (GNB2), β- and γ-actin, α- and β-tubulin, vimentin, neurofilament L, glial fibrillar acidic protein (GFAP), collapsin response mediator protein 2, CRMP2, DRP-2) (Pamplona et al., 2005), and 14-3-3 protein ζ (HYWAZ) and γ (HYWAG) isoforms (Santpere et al., 2007).

#### A redox model of alzheimer's disease pathogenesis

**Figure 3:**
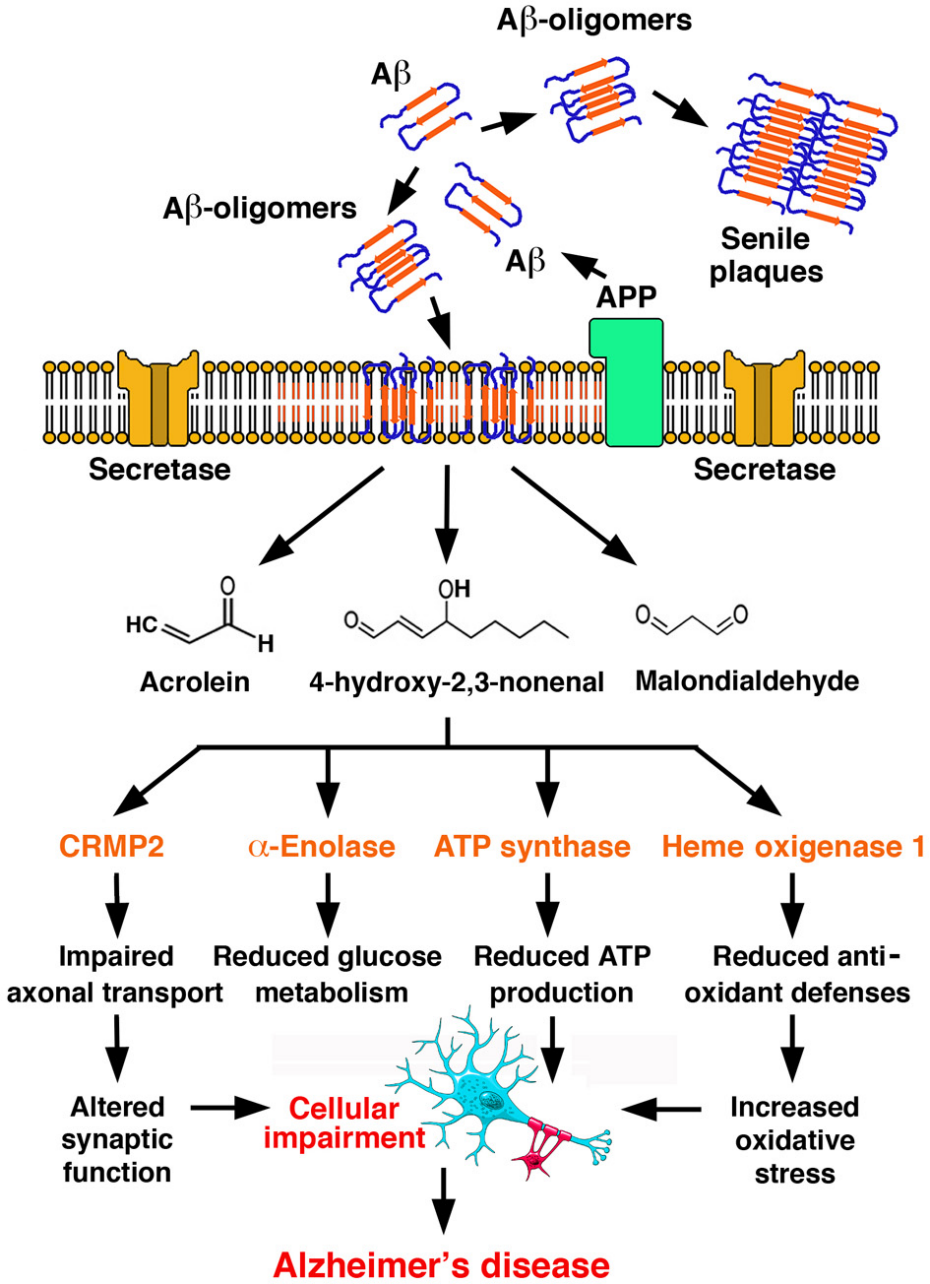
**A redox model of Alzheimer's disease pathogenesis**. Amyloid β-peptide (Aβ) is generated by proteolytic cleavage of Amyloid Precursor Protein (APP) by secretases. Aβ undergoes aggregation, with the formation of oligomers, which undergo a conformational transition to β-structured diffusible oligomers and eventually deposit as amyloid plaques in the ECM. Aβ oligomers insert in the plasma membrane, where they initiate lipid peroxidation, leading to the formation of reactive aldehydes, such as acrolein, MDA and HNE. Adduct formation compromises the function of critical proteins in a number of functional subsets of neurotransmission, energetic metabolism, mitochondrial function, antioxidant defenses, represented here by collapsin response mediated protein 2 (CRMP2), α-enolase, ATP synthase α subunit and heme oxygenase 1. Such process is self-feeding and ultimately leads to Alzheimer's disease [Redrawn with permission from Sultana et al. (2012)].

Far beyond individual protein dysfunctions, the generation of markers of lipid peroxidation in AD appears to be associated with the progressive endangerment of vital processes, such as energy metabolism, antioxidant defenses, signal transduction, axonal transport, and synapse conservation. A redox model of Alzheimer's disease pathogenesis (Sultana et al., 2012) is depicted in Figure 3.

## Aldehyde-protein adducts in atherosclerosis

The potential role of reactive aldehydes in the pathogenesis of atherosclerosis was suggested by their increases in plasma in association with extensive aortic atherosclerosis and the high levels of aldehydes generated during the oxidation of phospholipids in LDLs (Salomon et al., 2000). The observed consequences of LDL oxidation by aldehydes *in vitro* are described below. The formation of protein-bound lipid peroxidation products in atherosclerotic lesions was also repeatedly reported.

### Aldehyde-LDL adducts

Early studies of the contribution of aldehyde-protein adducts to atherogenesis provided evidences that modification of LDL by aldehydes enhanced their recognition and uptake by macrophages (Hoff et al., 2003). The formation of aldehyde adducts with apolipoprotein B (Apo B) in LDL converted the latter to an atherogenic form that was taken up by macrophages, leading to the formation of foam cells (Steinberg et al., 1989; Steinberg, 1995). The adduction products detected in Apo B of oxidized LDL included: (a) acrolein derivatives, such as N-(3-methylpyridinium)lysine (MP-Lys) (Obama et al., 2007) and the 3-formyl-3,4-dehydropiperidino adduct (FDP-lysine) formed by the addition of two acrolein molecules to one lysyl side chain (Uchida et al., 1998a,b); (b) HNE adducts, such as the enaminal-type HNE-histidine and HNE-lysine adducts (Uchida et al., 1994); (c) MDA adducts, such as Nε-(2-propenal)-lysine (Uchida et al., 1997), and 1-amino-3-iminopropene-type MDA-lysine cross-links (Requena et al., 1997). The formation of aldehyde-LDL adducts could alter the binding of LDL to membrane scavenger receptors at the surface of endothelial cells and activated macrophages. The participation of reactive aldehydes in LDL-receptor interactions was documented by several immunohistochemical analyses of atherosclerotic lesions from human aorta, using antibodies against various aldehyde adducts, such as HNE-histidine (Uchida et al., 1995), Nε-MDA-lysine (Uchida et al., 1997), and Nε-acrolein-lysine (FDP-lysine) (Uchida et al., 1998a), in which intense positivities were associated with cells, primarily macrophages. It was recently reported that HNE–histidine Michael adducts had significant affinities and interacted with LOX-1 (lectin-like oxidized low-density lipoprotein receptor-1), an important scavenger receptor mediating endothelial oxLDL uptake. HNE-modified proteins strongly inhibited the uptake of acetylated LDL (AcLDL). In human aortic endothelial cells, the binding of HNE-histidine adducts to LOX-1 stimulated ROS formation and activated extracellular signal-regulated kinase 1/2 (ERK 1/2) and NF-κB (Kumano-Kuramochi et al., 2012).

Using recombinant human Apo E (an exchangeable antiatherogenic apolipoprotein) and immunoblotting with acrolein-lysine-specific antibodies, other Authors (Tamamizu-Kato et al., 2007) showed that acrolein severely compromised the functional integrity of Apo E, as for heparin, lipid, and LDL receptor binding. These results were in agreement with previous observations of acrolein being widely present in atherosclerotic lesion, as revealed by the use of anti-acrolein antibodies (Uchida et al., 1998a). Nε-(3-methylpyridinium)-lysine (MP-Lys), an acrolein derivative, was detected in Apo B of native LDL (Obama et al., 2007). Moreover, acrolein-LDL induced foam cell formation from macrophages, suggesting that acrolein maight contribute to LDL modification, foam cell formation and atherogenesis (Watanabe et al., 2013).

### Phosphatidylcholine γ-hydroxyalkenals and atherosclerosis

Starting from the early observation that proteins modified by 2-pentylpyrrole incorporation of lysyl ε-amino groups, upon covalent addition of HNE, accumulated in the blood of individuals with atherosclerosis and in brain neurons of patients with Alzheimer's disease (Sayre et al., 1996), it became evident that γ-hydroxyalkenal phospholipids and their ω-carboxyalkylpyrrole derivatives contributed strongly in the pathogenesis of atherosclerosis. This was the subject of recent reviews (Salomon and Gu, 2011; Stemmer and Hermetter, 2012). Antibody-based studies revealed the presence of carboxyethylpyrroles (CHPs) and carboxypropylpyrroles (CPPs) in oxLDL (Kaur et al., 1997). Also the CHP immunoreactivity, reflecting the presence of protein adducts of 9-hydroxy-12-oxo-10-dodecenoic acid (HODA) or its phosphatidylcholine ester in human plasma, was significantly higher in the plasma of patients with atherosclerosis and end-stage renal disease than in healthy controls (Kaur et al., 1997). HODA-protein adducts were produced *in vivo* from 9-hydroxy-12-oxo-10-dodecenoyl-phosphatidylcholine (HODA-PC), one of the oxidized lipids derived from 1-palmityl-2-arachidonoyl-*sn*-glycero-3-phosphocholine (PA-PC), altogether referred to as oxPA-PC. Chemically synthesized 5-hydroxy-8-oxo-6-octenoyl-phosphatidylcholine (HOOA-PC) exhibited properties of a chemical mediator of chronic inflammation. It activated, in a dose-dependent manner, human aortic endothelial cells to bind monocytes and to secrete increased levels of monocyte chemotactic protein-1 (MCP-1) and interleukin-8 (IL-8), two chemokines promoting monocyte entry into chronic lesions. It also inhibited LPS-induced expression of E-selectin, an adhesion molecule mediating endothelial-neutrophil interactions (Subbanagounder et al., 2002). HOOA-PC was found unbound and in pyrrole adducts in lipid extracts of oxLDL and human atheromas (Podrez et al., 2002; Hoff et al., 2003). A scenario emerged from these studies, in which atherogenesis might involve myeloperoxidase-initiated, free radical-induced production of oxPC, which promoted subendothelial monocyte infiltration and endocytosis of oxLDL by macrophages, accompanied by conversion into foam cells and atheroma formation (Salomon and Gu, 2011). Thereafter, it was shown that scavenger receptor CD36, another mediator of oxLDL uptake by macrophages, at variance with the LDL receptor, bound oxidized lipid derivatives within oxLDL, including the derivatives of 1-palmitoyl-2-arachidonoyl-glycerophosphocholine (oxPA-PC), such as HOOA-PC, and of 1-linoleoyl-2-arachidonoyl-glycerophosphocholine (oxLA-PC), such as HODA-PC (Figure 2). These γ-oxygenated-α,β-unsaturated aldehydes, collectively referred to as oxPC_CD36_, were potent activators of the CD36-mediated endocytosis of oxLDL by macrophages, promoting the cytotoxic effects of the formation of protein adducts of electrophilic oxidized derivatives of cholesterol and phospholipids (Salomon and Gu, 2011). As an instance, the formation of Michael or pyrrole adducts of HOOA-PC or HODA-PC to a cysteinyl thiol group of lysosomal cathepsin B reduced the ability of mouse macrophages to degrade internalized macromolecules (Hoff et al., 2003). OxLDL and individual oxPC_CD36_ also interfered with the binding of HDL to scavenger SR-B1 receptors of hepatocytes, thus inhibiting the HDL-mediated delivery of cholesteryl esthers to the liver (Ashraf et al., 2008).

### HNE-scavenger receptor B1 adducts and keratinocyte HDL uptake

Scavenger Receptor B1 (SR-B1), also known as HDL receptor, is expressed in cells of the epidermal *stratum corneum*. In cultured human keratinocytes, exposure to cigarette smoke caused the translocation and eventual loss of SR-B1, driven by the activation of cellular NADPH oxidase (NOX) and the enhanced H_2_O_2_ production. Cigarette smoke also caused the formation of acrolein-SR-B1 and HNE-SR-B1 adducts and increased SR-B1 ubiquitination. It was proposed that such oxidation-dependent modifications of SR-B1 subcellular localization and stability might affect the physiological uptake of cholesterol by SC epidermal cells, which, in turn, might compromise their lipid composition and barrier function in the course of oxidative stress (Sticozzi et al., 2012).

## Aldehyde-protein adducts in autoimmunity

Modification of self antigens in the course of oxidative stress, by adduct formation with reactive products of lipid peroxidation, HNE being one of the most commonly involved, is generally regarded to as a mechanism by which concomitant modification of self and neoantigen formation may lead to the breaking of tolerance to self antigens and, thus, to the pathogenesis of autoimmune disease. Indeed, it was known for a long time that abnormally high levels of HNE-protein adducts can be detected in the sera of children affected by autoimmune diseases (Grune et al., 1997). According to this view, cross-linking of HNE with self antigens would be instrumental in creating neoantigens from formerly tolerated autoantigens and, thus, initiating autoimmunity.

### HNE-protein adducts in sjögren's syndrome (SS) and systemic lupus erythematosus (SLE)

Sjögren syndrome (SS) is an autoimmunity-driven chronic infammatory disorder, characterized by infiltration and destruction of lacrimal and salivary glands by effector CD4^+^ and CD8^+^ T cells and activated macrophages, resulting in keratoconjunctivitis with dry eyes and xerostomia (dry mouth). Secondary SS can also add to the clinical picture of other autoimmune diseases, such as systemic lupus erythematosus (SLE). Among autoimmune diseases, SS is second only to rheumatoid arthritis (RA) in prevalence (1%), with affected females outnumbering males by 9–1. Antibodies to self antigens, such as anti-nuclear antibodies (ANA), are characteristically found in SS, some of them being in common with other autoimmune diseases, such as SLE, RA, and systemic sclerosis (SSc). Typical ANA targets in SS include the SS-A/Ro and SS-B/La proteins. The former include a 52-kDa form, located in nucleus and in cytoplasm, (SS-A1/Ro52; TRIM21) and a 60-kDa cytoplasmic form (SS-A2/Ro60; TROVE2). Both are components of Ro ribonucleoprotein (RNP) particles, in which they associate with short non-coding, histidine-rich RNAs (hY-RNAs). The 48-kDa SS-B/La antigen is a transcription termination factor for RNA Polymerase III, transiently associated with hY-RNAs in ribonucleoprotein particles involved in tRNA processing and histonic mRNA stabilization. Systemic lupus erythematosus (SLE) is a multisystemic disease characterized by a polyclonal B cell activation, leading to the differentiation of plasma cells producing autoantibodies toward a broad range of autoantigens. ANA are found in 95% of patients with SLE, as well as in patients with other autoimmune diseases. They are heterogeneous and include antibodies toward: double stranded (ds) DNA; histones; ribonucleoproteins (RNP), such as the Smith (Sm) antigen (corresponding to the common core proteins of spliceosomal small nuclear RNPs), and the SS-A/Ro and SS-B/La antigens. The formation and deposition of immune complexes and complement in the wall of small arteries, at the dermo-epidermal junction and in the glomerular basal membrane (GBM) is responsible, respectively, for the diffuse necrotizing vasculitis, the cutaneous lesions of erythematous, bullous, and ulcerative kind, and the nephritis associated with SLE.

Notwithstanding their nuclear and/or cytoplasmic location, Ro and La antigens appeared to become exposed at the cell surface in the course of apoptosis. Epitopes expressed at the surface of apoptotic cells are named “apotopes.” After the first observations of the clustering of cytoplasmic and nuclear antigens, including SS-Ro and SS-La antigens, in two types of blebs at the surface of apoptotic cells (Casciola-Rosen et al., 1994), the accessibility of SS-A/Ro and SS-B/La antigens at the surface of apoptotic cells was further confirmed (Miranda-Carús et al., 2000; Ohlsson et al., 2002). In SLE and SS, both the number of circulating apoptotic leukocytes and the susceptibility of lymphocytes to activation-induced apoptosis *in vitro* increased (Emlen et al., 1994; Georgescu et al., 1997; Zeher et al., 1999; Ren et al., 2003). Impaired efferocytosis (clearance of apoptotic cells) by macrophages also contributed to the higher degree of exposure to autoantigens determined by the increased rate of apoptosis in SLE (Ren et al., 2003). It was speculated that both factors may trigger autoimmunity (Savill et al., 2002). It was proposed (Casciola-Rosen et al., 1994) that the breaking of tolerance to autoantigens at the surface of apoptotic cells might be favored by oxidative modifications occurring as a result of the oxidative stress that characterizes apoptosis (Hockenberry et al., 1993).

The contribution of the formation of HNE adducts to the modification of self antigens, such as SS-A2/Ro60, in Sjögren's syndrome was explored by Scofield and coworkers. They hypothesized that modification of SS-A2/Ro60 with HNE might facilitate the breaking of tolerance to the self antigen. After immunizing rabbits with either HNE-modified or unmodified SS-A2/Ro60, they observed that autoimmunity was established faster and more strongly in animals immunized with HNE-modified SS-A2/Ro60 (Scofield et al., 2005). In an extension of this model, an SS-like condition, with anti-SS-A2/Ro60 antibodies, decreased salivary flow and salivary gland mononuclear infiltrates, could be induced in BALB/c mice by immunization with a peptide of SS-A2/Ro60 (Kurien et al., 2011). Efficient production of anti-SS-A2/Ro60 and anti-SS-B/La autoantibodies ensued immunization with SS-A2/Ro60, both as such and modified with increasing concentrations of HNE (0.4, 2, or 10 mM). However, antibody production was faster after low- and medium-level modification of SS-A2/Ro60 with HNE. Differential use of unmodified or HNE-modified SS-A2/Ro60 as the solid-phase substrate in ELISAs for autoantibodies revealed, among the antibodies produced by mice immunized with HNE-modified SS-A2/Ro60, an additional subpopulation of antibodies, which recognized HNE or HNE-SS-A2/Ro60, but not unmodified SS-A2/Ro60. Most interestingly, immunization with medium-level HNE-modified SS-A2/Ro60 was accompanied by the appearance of anti-dsDNA autoantibodies, which induced the Authors to imply a SLE-like disease, although they did not provide pathological evidence of it. Together with the already mentioned appearance of anti-SS-B/La antibodies, following immunization with SS-A2/Ro60, the occurrence of anti-dsDNA antibodies represented an example of intermolecular epitope spreading. In turn, the ability of HNE to form adducts with a large number of biological macromolecules could be of help in understanding the broad range of autoantibody responses in SLE and SS. Moreover, immunization with high-level HNE-modified SS-A2/Ro60 was associated with weaker antibody responses to unmodified SS-A2/Ro60 and SS-B/La, reduction of salivary flow and lymphocytic infiltration of salivary glands, suggesting a Sjögren's syndrome-like condition. Notably, high-level HNE modification of SS-A2/Ro60 was accompanied by aggregation, which prompted the Authors to interpret the results as due to increasing bifunctional cross-linking of SS-A2/Ro60 and diminished exposure of HNE at the surface of SS-A2/Ro60 molecules (Kurien et al., 2011). A more likely interpretation could be that large, particulate immunocomplexes of aggregated HNE-SS-A2/Ro60 and autoantibodies stimulated the phagocytic and antigen-presenting activity of macrophages, which skewed the autoimmune response toward a prevailingly cytotoxic cell-mediated mechanism.

The molecular mimicry between the adducts of lipid peroxidation products with proteins and nucleic acids, as a possible mechanism initiating the production of anti-DNA autoantibodies, in response to some other modified self antigen, was the subject of interesting studies by Uchida and coworkers. After raising an anti-HNE monoclonal antibody (anti-*R* mAb 310), recognizing enantioselectively (*R*)-HNE-histidine Michael adducts (Hashimoto et al., 2003), they unexpectedly found that the sequence of this anti-HNE mAb was highly similar to those of various clonally related anti-DNA antibodies. Despite these structural similarities, the cross-reactivity of mAb R310 with native dsDNA was limited, but was strongly enhanced by treating DNA with 4-oxo-2-nonenal (ONE), a HNE analog. The 7-(2-oxo-heptyl)-substituted 1,*N*^2^-etheno-type ONE-2'-deoxynucleoside adducts were identified as alternative epitopes of mAb R310 in ONE-modified DNA. On these grounds, these Authors hypothesized that endogenous reactive electrophiles, like HNE, might function as immunologic triggers for human autoimmunity (Akagawa et al., 2006). These Authors further investigated the possible involvement of HNE-modified proteins as the endogenous source of anti-DNA antibodies. They found HNE-specific epitopes in the epidermis and dermis of patients with SLE, pemphigus vulgaris and contact dermatitis, as well as antibodies against HNE-modified bovine serum albumin (BSA) in the sera of patients affected with SLE, Siögren's syndrome, rheumatoid arthritis, systemic sclerosis and idiopathic inflammatory miopathies, and also in the sera of diseased MRL/*lpr* mice. Upon repeated immunization with HNE-modified KLH, mice developed also a subpopulation of B cell clones recognizing native DNA, but not HNE-BSA. In agreement with previous results, the reactivity of anti-HNE B cell clones toward DNA was greatly enhanced by DNA modification with ONE. On the other hand, anti-DNA mAbs cross-reacted with ONE-modified BSA. These data suggested that HNE-specific epitopes produced upon physiological generation of HNE in cells might serve as triggering antigens for the development of bispecific antibodies against native DNA and ONE-modified proteins. On the whole, these findings strongly supported the pathogenic role of lipid peroxidation products in autoimmune disease (Toyoda et al., 2007). The pathogenic role of lipid peroxidation in SLE and the potential usefulness of anti-MDA and anti-HNE antibody titers in predicting its progression was underscored also by a report showing that the prevalences and serum levels of MDA- and HNE-protein adducts, as well as of MDA- and HNE-specific antibodies, were significantly higher in SLE patients than in healthy controls, and were in correlation with the SLE Disease Activity Index. The levels of each aldehyde-protein adduct were also in correlation with the titers of the respective antibodies (Wang et al., 2010).

## Aldehyde-protein adducts and structural integrity, ion transport, and signal transduction at the plasma membrane level

HNE is the product of lipid peroxidation which has been shown to be mostly involved in the control of cell functions. Under physiological conditions, HNE can be found at low concentrations in human tissues and plasma (Parola et al., 1998; Okada et al., 1999; Ji et al., 2001; Siems and Grune, 2003), where it participates in the control of signal transduction, cell proliferation and differentiation (Parola et al., 1998). HNE-protein adducts in peripheral blood primarily involve albumin, transferrin and immunoglobins (Barrera et al., 1996). Adducts between HNE and proteins have been detected *in vitro* in various mammalian cell types (Parola et al., 1998; Okada et al., 1999; Ji et al., 2001; Siems and Grune, 2003), in which the percent of total added HNE in HNE-protein adducts was between 1 and 5% (Rinaldi et al., 2001). Some adducts of HNE with cell proteins involved in specific functions at the plasma membrane level were characterized in detail.

### HNE-spectrin adducts and red cell membrane integrity

Spectrin is the main component of the submembranous cytoskeleton lining the intracellular side of the plasma membrane of red blood cells, playing a fundamental role in maintaining its stability and strength, via direct interactions with membrane lipids and the actin cytoskeleton. Immunoblotting and mass spectrometric analyses revealed that, in human red cells, α- and β-spectrin were the primary targets of HNE adduction. Exposure of intact red cells to HNE resulted in selective HNE-spectrin adduct formation, with preferential β-spectrin modification and cross-linking of HNE-modified spectrin molecules. The Authors speculated that local spectrin aggregation, by freeing the lipid bilayer from the underlying spectrin-actin cytoskeleton, might lead to membrane surface area loss by extrusion (Arashiki et al., 2010). Together with the reported accumulation of HNE in aging circulating red blood cells (Ando et al., 1995), these observations may be of relevance not only for the physiological destruction of aged red cells, but also for the immune-mediated hemolysis of red blood cells under conditions of enhanced production of lipid hydroperoxides.

### HNE-Na^+^-K^+^-ATPase adducts

Na^+^-K^+^-ATPase is an integral plasma membrane protein of great functional importance. Its primary functions are the maintenance of intracellular K^+^ ion levels and the excretion of Na^+^ ions. It contains 70 cysteinyl residues per molecule. The binding of HNE at 1–10 μmolar concentration to Na^+^-K^+^-ATPase was rapid and was accompanied by a decrease in measurable SH- groups and an irreversible loss of enzyme activity (Siems et al., 1996). Na^+^-K^+^-ATPase could be attacked by HNE formed both intra- and extracellularly, due to the free access of HNE to integral plasma membrane proteins. These Authors suggested that the reduction of Na^+^-K^+^-ATPase activity upon covalent HNE binding might represent an important form of secondary oxidative cell damage. Their findings were confirmed by the demonstration that in cultured hippocampal neurons HNE impaired Na^+^-K^+^-ATPase activity and induced increases of intracellular Ca^2+^ ion concentration (Mark et al., 1997b).

### HNE adducts with tyrosine kinase receptors

Tyrosine kinase receptors (RTKs), such as the epidermal growth factor receptor (EGFR) and the platelet-derived growth factor receptor (PDGFR), are transmembrane glycoproteins, displaying tyrosine kinase activity in their cytoplasmic domains. Stimulation of RTKs by ligand-dependent or -independent mechanisms (radiation, metal ions, ROS) induces receptor dimerization and autophosphorylation of tyrosyl residues, followed by catalytic activation, whereas downregulation of RTKs is mediated by internalization and dephosphorylation (Pawson and Scott, 1997). Oxidized LDL (but not native LDL) and free HNE induced in living cells the formation of HNE-EGFR and HNE–PDGFR adducts, evidenced by the binding of anti-HNE-protein antibodies and by the loss in free −NH_2_ group content (Suc et al., 1998; Hubbard and Till, 2000; Escargueil-Blanc et al., 2001). At physiological or moderate HNE concentrations (0.1 μ M and 1–10 μ M), the formation of HNE-EGFR and HNE-PDGFR adducts resulted in sustained RTK activation (Suc et al., 1998; Escargueil-Blanc et al., 2001). A short incubation of vascular smooth muscle cells (SMCs) with a low concentration of HNE (0.1–1 μ M) induced the derivatization and autophosphorylation of RTKs, with the consequential activation of the phosphatidylinositol 3-kinase (PI3K)/Akt-mediated survival pathway and of the mitogenic response of SMCs (Auge et al., 2002). On the other hand, high concentrations of HNE, for longer incubation times, inhibited EGFR- and PDGFR-mediated cell proliferation (Liu et al., 1999; Vindis et al., 2006), through inhibitory effects on RTK signaling (Negre-Salvayre et al., 2003). High doses of HNE exerted similar negative effects on proteasomes (Okada et al., 1999; Vieira et al., 2000), mitochondrial transition pores (Irwin et al., 2002), glyceraldehyde-3-phosphate dehydrogenase (Uchida and Stadtman, 1993) and cathepsin B activities (Crabb et al., 2002). The inhibitory effect of 4-HNE on growth factor-dependent cell proliferation was in agreement with the progressive desensitization of PDGFR β subunit to its ligand PDGF B-chain in SMCs (Vindis et al., 2006). In other cell types, low HNE concentrations (1 μ M) did not cause RKT activation. In human hepatic stellate cells (hHSC), 1 μ M HNE rather inhibited tyrosine autophosphorylation of PDGFR β induced by the PDGF BB isoform, which resulted in the inhibition of the mitogen-activated protein kinase (MAPK) and PI3K cascades and a consequential decrease of PDGF-dependent DNA synthesis (Robino et al., 2000). Acrolein was also shown to be a potent inactivator of protein tyrosine phosphatase 1B (PTP1B), a member of an important class of cysteine-dependent enzymes, working in tandem with protein tyrosine kinases in the regulation of a number of signal transduction pathways (Seiner et al., 2007).

### HNE adducts with proteins in the insulin signaling cascades

The regulation of insulin signaling starts with the binding of insulin to its receptor, whose tyrosyl residues are rapidly phosphorylated. This permits the recruitment of adaptor proteins, such as insulin receptor substrates (IRSs) and Src homology-2-containing (Shc) proteins, which transmit the insulin signal down the PI3K cascade for glucose, lipid, and protein metabolism and the MAPK cascade for cell proliferation and differentiation (Saltiel and Kahn, 2001; Van Obberghen et al., 2001; White, 2002; Taniguchi et al., 2006). Reductions in the levels of IRSs and insulin-induced IRSs and a decrease in insulin receptor β phosphorylation were observed upon exposure to HNE at non-toxic concentrations (Demozay et al., 2008). Such effects could be due to the formation of HNE-IRS adducts, likely impairing IRS function and favoring IRS degradation. The downstream signaling cascades, involving PI3K and protein kinase B (PKB), were also down-regulated upon exposure to HNE, which resulted in blunted metabolic responses. The Authors of this study hypothesized that HNE build-up in diabetic rats (due to increased lipid peroxidation and altered clearance of its products by detoxifying enzymes) might be a cause of signaling dysfunction, hindering insulin action.

## Conclusions

The adducts of reactive aldehydes with membrane proteins participate in physiological, as well as pathological processes and can determine variable functional consequences, in relation with the protein targets of adduction and their functional roles. Polyclonal and monoclonal antibodies directed against protein-bound aldehyde adducts have been of great help in exploring the aldehyde-related modifications of the cell proteome, while mass spectrometry-based techniques have been playing a key role in elucidating the stoichiometry and sites of covalent protein modification with reactive aldehydes. Nonetheless, the inventory of aldehyde-modified membrane proteins detected so far is probably still largely incomplete, when compared with the plethora of biological effects displayed by these molecules. Quantitative technical limitations in the individuation of aldehyde-protein adducts are being gradually overcome by the increases in sensitivity, molecular specificity and tolerance to impurities of spectrometric instrumentation and techniques (Wu and Vogt, 2012). Current challenges include: (1) characterizing the functional consequences of cell protein modification with aldehydes, which was not addressed by most redox proteomic studies published until now. This may involve major efforts of expression, reconstitution, modification and activity/interactivity assays of protein targets of aldehyde modification *in vitro*, as well as innovative approaches of protein-specific tracking and functional characterization at the cellular level; (2) clarifying the sources, sites and circumstances of increased lipid peroxidation in cells and the topological/functional relationships (e.g., in terms of subcellular compartmentalization and regulation of gene expression and gene product activity) linking the increased generation of reactive aldehydes with the modifications of specific cell membrane proteins.

### Conflict of interest statement

The authors declare that the research was conducted in the absence of any commercial or financial relationships that could be construed as a potential conflict of interest.
