# Supplementary material for: Interaction of aldehydes derived from lipid peroxidation and membrane proteins
Source: Front Physiol. 2013 Sep 4;4:242. doi: 10.3389/fphys.2013.00242 (PMC3761222; doi:10.3389/fphys.2013.00242)
Supplement: Supplementary file 1 [file DataSheet1.PDF]

Home | Account Info | Help

Title: Discovery of Carboxyethylpyrroles (CEPs): Critical Insights into AMD, Autism, Cancer, and Wound Healing from Basic Research on the Chemistry of Oxidized Phospholipids

Author: Robert G. Salomon, Li Hong, and Joe G. Hollyfield

Publication: Chemical Research in Toxicology

Publisher: American Chemical Society

Date: Nov 1, 2011

Copyright © 2011, American Chemical Society

Logged in as:
Fabrizio Gentile
Account #:
3000676518

LOGOUT

PERMISSION/LICENSE IS GRANTED FOR YOUR ORDER AT NO CHARGE

This type of permission/license, instead of the standard Terms & Conditions, is sent to you because no fee is being charged for your order. Please note the following:

- Permission is granted for your request in both print and electronic formats, and translations.
- If figures and/or tables were requested, they may be adapted or used in part.
- Please print this page for your records and send a copy of it to your publisher/graduate school.
- Appropriate credit for the requested material should be given as follows: "Reprinted (adapted) with permission from (COMPLETE REFERENCE CITATION). Copyright (YEAR) American Chemical Society." Insert appropriate information in place of the capitalized words.
- One-time permission is granted only for the use specified in your request. No additional uses are granted (such as derivative works or other editions). For any other uses, please submit a new request.

If credit is given to another source for the material you requested, permission must be obtained from that source.

BACK | CLOSE WINDOW

Copyright © 2013 Copyright Clearance Center, Inc. All Rights Reserved. Privacy statement.
Comments? We would like to hear from you. E-mail us at customercare@copyright.com
