# Supplementary material for: Interaction of aldehydes derived from lipid peroxidation and membrane proteins
Source: Front Physiol. 2013 Sep 4;4:242. doi: 10.3389/fphys.2013.00242 (PMC3761222; doi:10.3389/fphys.2013.00242)
Supplement: Supplementary file 2 [file DataSheet2.PDF]

ELSEVIER LICENSE
TERMS AND CONDITIONS

Jul 16, 2013

This is a License Agreement between Fabrizio Gentile ("You") and Elsevier ("Elsevier") provided by Copyright Clearance Center ("CCC"). The license consists of your order details, the terms and conditions provided by Elsevier, and the payment terms and conditions.

All payments must be made in full to CCC. For payment instructions, please see information listed at the bottom of this form.

Supplier	Elsevier Limited<br>The Boulevard,Langford Lane<br>Kidlington,Oxford,OX5 1GB,UK
Registered Company Number	1982084
Customer name	Fabrizio Gentile
Customer address	DiMeS - Via De Sanctis, snc
	Campobasso, CB 86100
License number	3190740330452
License date	Jul 16, 2013
Licensed content publisher	Elsevier
Licensed content publication	Free Radical Biology and Medicine
Licensed content title	Lipid peroxidation triggers neurodegeneration: A redox proteomics view into the Alzheimer disease brain
Licensed content author	Rukhsana Sultana,Marzia Perluigi,D. Allan Butterfield
Licensed content date	5 October 2012
Licensed content volume number	
Licensed content issue number	
Number of pages	1
Start Page	0
End Page	0
Type of Use	reuse in a journal/magazine
Requestor type	author of new work
Intended publisher of new work	Other

Print This Page
